# Immune-Associated Gene Signatures Serve as a Promising Biomarker of Immunotherapeutic Prognosis for Renal Clear Cell Carcinoma

**DOI:** 10.3389/fimmu.2022.890150

**Published:** 2022-05-24

**Authors:** Qi Wang, Hanmin Tang, Xuehui Luo, Jie Chen, Xinyue Zhang, Xinyue Li, Yuesen Li, Yuetong Chen, Yungang Xu, Suxia Han

**Affiliations:** ^1^ Department of Radiation Oncology, First Affiliated Hospital of Xi’an Jiaotong University, Xi’an, China; ^2^ Department of Cell Biology and Genetics, School of Basic Medical Sciences, Xi’an Jiaotong University Health Science Center, Xi’an, China

**Keywords:** renal clear cell carcinoma (ccRCC), immune checkpoint inhibition, IRPDGs, tumor microenvironment, prognostic biomarker

## Abstract

As the most common type of renal cell carcinoma (RCC), the renal clear cell carcinoma (ccRCC) is highly malignant and insensitive to chemotherapy or radiotherapy. Although systemic immunotherapies have been successfully applied to ccRCC in recent years, screening for patients who can benefit most from these therapies is still essential and challenging due to immunological heterogeneity of ccRCC patients. To this end, we implemented a series of deep investigation on the expression and clinic data of ccRCC from The Cancer Genome Atlas (TCGA) International Consortium for Cancer Genomics (ICGC). We identified a total of 946 immune-related genes that were differentially expressed. Among them, five independent genes, including SHC1, WNT5A, NRP1, TGFA, and IL4R, were significantly associated with survival and used to construct the immune-related prognostic differential gene signature (IRPDGs). Then the ccRCC patients were categorized into high-risk and low-risk subgroups based on the median risk score of the IRPDGs. IRPDGs subgroups displays distinct genomic and immunological characteristics. Known immunotherapy-related genes show different mutation burden, wherein the mutation rate of VHL was higher than 40% in the two IRPDGs subgroups, and SETD2 and BAP1 mutations differed most between two groups with higher frequency in the high-risk subgroup. Moreover, IRPDGs subgroups had different abundance in tumor-infiltrating immune cells (TIICs) with distinct immunotherapy efficacy. Plasma cells, regulatory cells (Tregs), follicular helper T cells (Tfh), and M0 macrophages were enriched in the high-risk group with a higher tumor immune dysfunction and rejection (TIDE) score. In contrast, the low-risk group had abundant M1 macrophages, mast cell resting and dendritic cell resting infiltrates with lower TIDE score and benefited more from immune checkpoint inhibitors (ICI) treatment. Compared with other biomarkers, such as TIDE and tumor inflammatory signatures (TIS), IRPDGs demonstrated to be a better biomarker for assessing the prognosis of ccRCC and the efficacy of ICI treatment with the promise in screening precise patients for specific immunotherapies.

## Introduction

Renal cell carcinoma (RCC) is one of the most common malignancies in humans. It is divided into renal papillary cell carcinoma, renal clear cell carcinoma (ccRCC), and chromophobe renal cell carcinoma. ccRCC is the most common type, accounting for over 80% of renal cancers (1). ccRCC is highly malignant and insensitive to chemotherapy or radiotherapy. At present, surgical resection is an effective treatment for early limited ccRCC. However, approximately 25% to 50% of primary patients experience recurrence five years after nephrectomy, and one-third of patients develop metastases; the overall 5-year survival rate is only 14% (1–3). ccRCC has distinct immunological features, such as a high degree of immune infiltration, relatively low mutational load, and relative sensitivity to anti-angiogenic therapy and immunotherapy (4).

Systemic immunotherapy has been successfully applied to ccRCC in recent years and has shown great benefits. Cytokine-based immunotherapy, including interleukin 2 (IL-2) and recombinant human interferon-alpha 2a (IFN-α2a), is the first approved immunotherapy for renal cell carcinoma. The combined results of several studies have shown an overall objective response rate (ORR) of 14% in IL-2-treated ccRCC patients, with 5% having complete response (CR) and 9% partial response (PR) (5). IFN-α2a treatment has an ORR of 7.5%–10% in patients with advanced ccRCC (6, 7). The ORR of both cytokine treatments is generally low and is accompanied by significant toxicity to multiple major organs (2). Moore et al. discovered that the Von Hippel-Lindau (VHL) gene is mutated in more than 80% of renal cell carcinomas owing to promoter methylation, resulting in VHL protein dysfunction, which in turn induces hypoxia-inducible factor (HIF) activation and leads to abnormal expression of downstream vascular endothelial growth factor (VEGF) (8–10). After this discovery, tyrosine kinase inhibitors (TKIs), such as sunitinib and cabozantinib, which target the VEGF pathway and neovascularization, have been progressively used in ccRCC. The ORR of sunitinib monotherapy was 27%; however, previous studies have reported grade 3 or higher adverse reactions in 65%–70.6% of patients (11–13). Although the efficacy of TKIs over previous cytokine therapy has been confirmed, most patients with ccRCC develop drug resistance within one year (14).

The advent of immune checkpoint inhibitors (ICI) has solved the problems of low response rate and high toxicity in both cytokine and TKI treatments, and they are now the primary treatment option for ccRCC in clinical settings. Previous studies have demonstrated that immune checkpoints are involved in regulating cytotoxic T lymphocyte (CTL) activation and effector function to maintain autoimmune tolerance, and tumor cells evade the body’s immunosurveillance in this way (15). ICI therapy achieves the antitumor effect by reactivating the immune response effect of CTL. ICIs currently used in the treatment of ccRCC mainly include pembrolizumab and nivolumab (PD-1 suppressants); avelumab and atezolizumab (PD-L1 suppressants); and ipilimumab (CTLA-4 suppressant). The ORR improves to 20%–42% with ICI monotherapy (13, 16–18). With the approval of TKIs and ICI in combination for ccRCC, multiple phase-III trials have provided many combination options of ICI and TKIs for clinical first- and second-line immunotherapy, with an ORR of 39.1%–71%. However, previous studies have reported that more than 90% of patients receiving the combination treatment of TKI and ICI had adverse events, of which 46%–82.4% had grade 3 or higher adverse events (13, 19–25). Although the ORR of ICI monotherapy is not satisfactory, the combination of ICI and TKIs improves the ORR to some extent, but also increases toxicity.

Screening for patients who can benefit most from these therapies is essential. Several studies have found that many factors, including tumor microenvironment (TME), influence the efficacy of ICI treatment (26, 27). Tumor-infiltrating immune cells (TIICs) in the TME of ccRCC play a key role in both pro- and anti-tumor processes, and are closely associated with clinical regression and response to immunotherapy (8, 10, 28, 29). According to Gulati et al., the TME of ccRCC is extremely heterogeneous. Patients with the same degree of progression may show different treatment responses and prognoses when receiving immunotherapy (30). Thus, there is a need to elucidate the interactions of various TIICs in the TME and to screen patients who are more suitable for immunotherapy. Current clinical treatment options usually rely on the International Metastatic Kidney Cancer Database (IMDC) or Memorial Sloan-Kettering Cancer Center (MSKCC) criteria (31, 32). The prognosis of ccRCC is primarily based on pathological staging (33); however, the current pathological staging system is inadequate to identify patients who are prone to adverse effects and low response rate when receiving ICI therapy. To advance the individualization of immunotherapy, it is urgent to discover new potential biomarkers to predict clinical response to ICI and prognosis.

**Figure 1:**
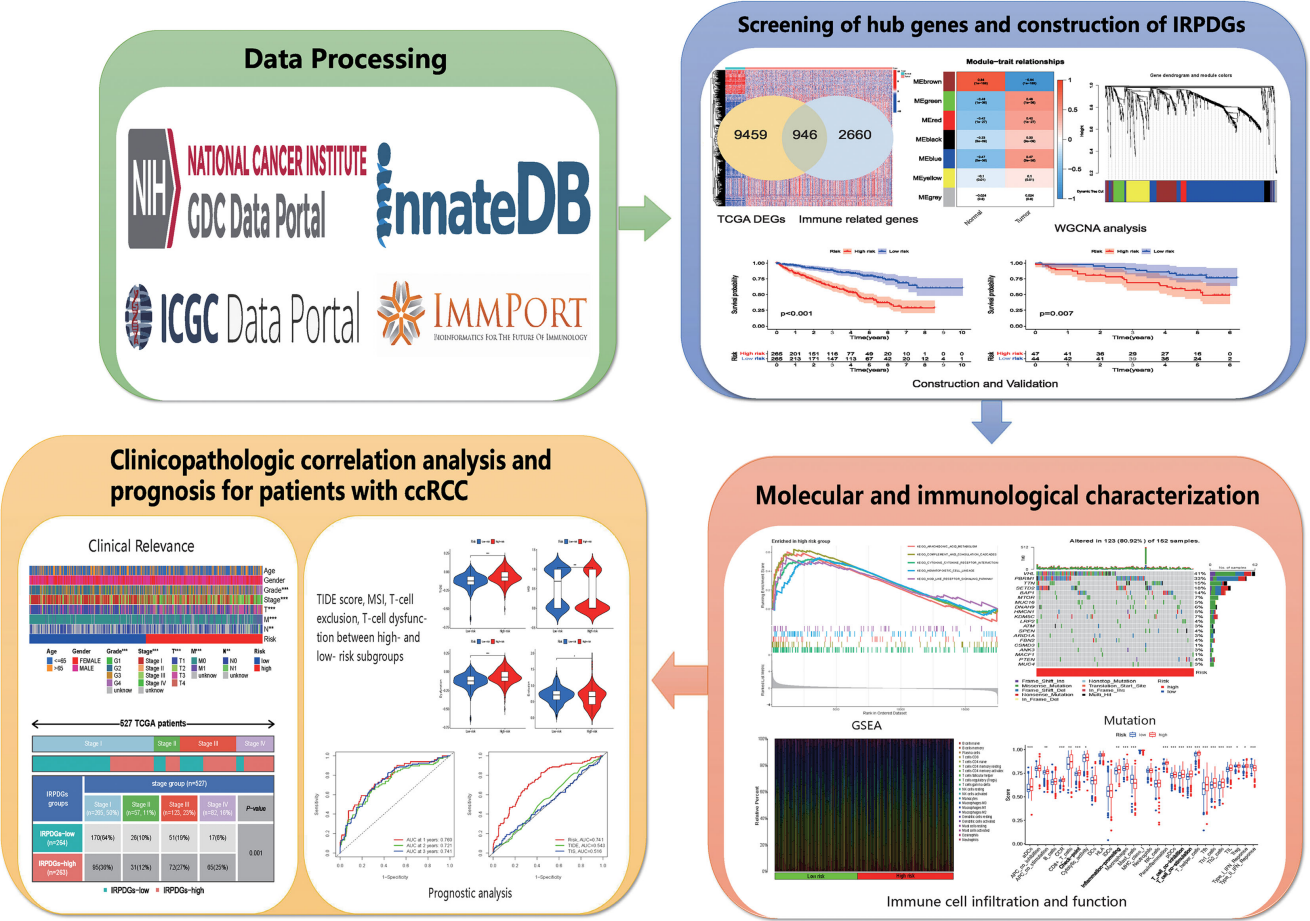
Workflow of the study.

In this study, we collected KIRC-mRNA data and related clinical information for ccRCC from TCGA. We initially screened 24 immune-related hub genes by applying WGCNA and univariate Cox regression analysis. Then, we constructed an independent immune-related prognostic differential gene signature (IRPDGs) containing five genes using multivariate Cox regression analysis. Data from ICGC were used for external validation. We defined high- and low-risk subgroups of the IRPDGs based on the median risk score and used gene set enrichment analysis (GSEA) to explore important signaling pathways and potential molecular mechanisms enriched in the IRPDGs subgroups. We also downloaded the corresponding mutation data in TCGA for a comprehensive analysis of somatic mutations. We used the deconvolution algorithm CIBERSORT (34) and single sample genes set enrichment analysis (ssGSEA) to analyze the relative infiltration abundance of 22 different TIICs and 29 immune-associated functional indicators in different subgroups. Receiver operating characteristic (ROC) analysis was performed to further assess the prognostic value of the IRPDGs. Compared with other biomarkers such as tumor immune dysfunction and rejection (TIDE) and 18-gene tumor inflammatory signatures (TIS), the IRPDGs demonstrated significant advantages in predicting the prognosis of patients treated with immunotherapy. The workflow for this study is shown in **Figure 1**.

## Materials and Methods

### Patients and Data

We downloaded RNA-sequencing data and detailed clinical information for ccRCC from TCGA (https://portal.gdc.cancer.gov/), which contains 539 cancer samples and 72 paracancerous samples. A total of 91 specimens were obtained from the ICGC database (https://dcc.icgc.org/releases) for external validation. Perl language (version 5.30.2) was used to extract clinical information. The list of immune-related genes was obtained from the Immport (https://www.Immport.org/home/) and the InnateDB (https://www.innateDBdb.com/) databases.

### Search for Immune-Related Hub Genes

The DEGs were screened from 539 ccRCC samples and 72 paracancerous samples with the LIMMA package (p-value < 0.05, |log2FC| > 1), and were intersected with the downloaded list of the immune-related genes to obtain immune-related DEGs. The Gene Ontology (GO) and the Kyoto Encyclopedia of Genes and Genomes (KEGG) were used to explore the major pathways enriched in immune-related hub genes. We used WGCNA (35) to identify hub genes. We first calculated Pearson correlation coefficients, constructed a similarity matrix using the expression data, and converted the similarity matrix into a neighborhood matrix with a soft threshold of 3; we then built TOM matrix based on the neighborhood matrix, clustered genes with a distance of 1−TOM, and identified and constructed different gene modules for visualization. Seven modules were obtained, and the top two modules (brown and green modules) with the most significant correlations were chosen. We filtered the top 40 core genes in the two modules by using CytoHuba (36). Finally, the best cutoff values of the core genes were calculated by the R package, and a total of 24 immune-related hub genes closely related to ccRCC survival were screened for further analysis.

### Construction and Validation of IRPDGs

Five genes independently and significantly associated with ccRCC prognosis were screened from 24 hub genes by multivariate Cox regression analysis. The IRPDGs was defined as the result of multiplying the expression value of each gene separately by the sum of the weights in the Cox model. Different subgroups of IRPDGs were defined according to the median risk score. The IGCG data (n = 91) were used for validation. Kaplan–Meier survival analysis with log-rank tests was performed to assess the prognostic power of the IRPDGs (P < 0.05).

### Molecular Profiling in the IRPDGs Subgroups

The GSEA was used to reveal important signaling pathways and potential molecular mechanisms enriched in different subgroups. A reference genome (c2.Cp.kegg.v7.4.Symbols.Gmt) was taken to compare immunophenotypes between the IRPDGs subgroups (with p < 0.05 and FDR < 0.25 as cutoff values). We downloaded mutation information associated with ccRCC from TCGA, and the Maftools package in the R software was used to comprehensively analyze somatic mutations between the IRPDGs subgroups.

### Assessment of Immune Characteristics and Immunotherapy

Based on ccRCC gene expression profiles, the abundance of 22 TIICs in different subgroups was quantified by using CIBERSORT (https://CIBERSORT.stanford). We used the GSVA package for ssGSEA of the enrichment levels of 29 immune-related functional indicators. Using the “survival ROC” R-package for survival analyses, we calculated areas under the curve (AUC) at different cutoff time points. To further explore the value of the IRPDGs in prognosis, we analyzed differences in TIDE score, microsatellite instability (MSI), T-cell dysfunction, and T-cell rejection effects in the IRPDGs subgroups. We compared the prognostic ability of the IRPDGs, TIDE, and TIS. The TIDE score was calculated online (http://tide.dfci.atherard.edu/), and the TIS score was defined as the mean of the log2-scaled normalized expression of 18 marker genes (37).

### Statistical Analysis

R program was used in most of our research. Pearson correlation tests were used to analyze the characteristics of immune-related hub genes. Differences between variables were analyzed using independent t-tests and chi-square tests. The prognostic value of the IRPDGs compared with clinicopathological features of ccRCC was assessed by the K–M survival and Cox regression analysis. Two-tailed P < 0.05 was considered significant.

## Results

### Screening of Immune-Related Hub Genes

**Figure 2:**
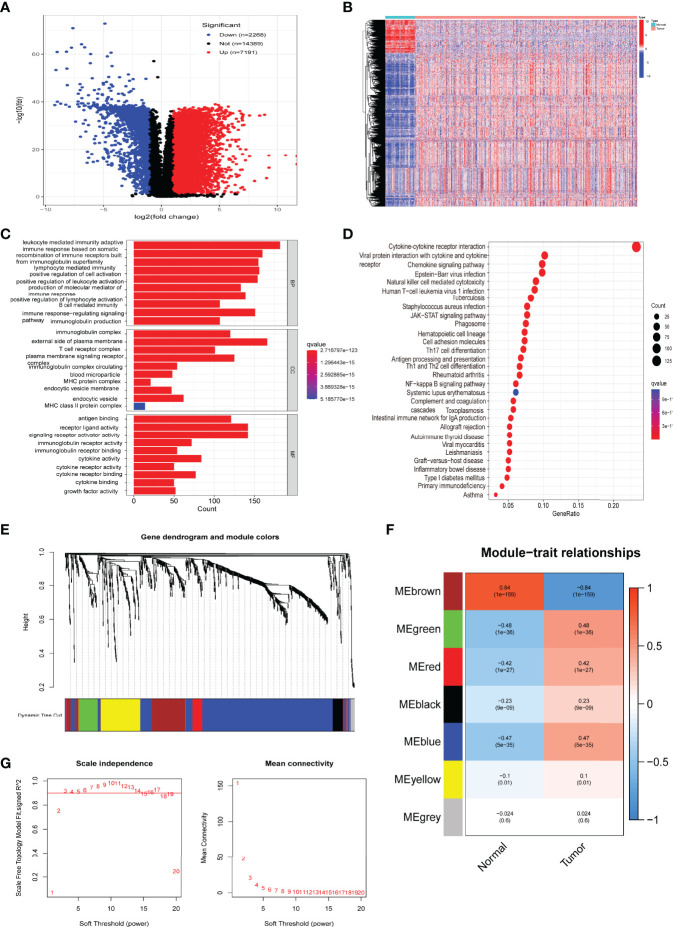
Screening and functional enrichment analysis of immune-related differentially expressed genes (DEGs), with identification of hub genes. **(A)** Volcano plot of ccRCC differentially expressed genes. **(B)** Heatmap of immune-related DEGs. **(C)** Gene Ontology (GO) enrichment analysis of immune-related DEGs. **(D)** Kyoto Encyclopedia of Genes and Genomes (KEGG) pathway analysis of immune-related DEGs. **(E)** Gene dendrograms and color distribution were obtained from WGCNA. **(F)** Gene modules with different survival relevance were obtained by WGCNA. **(G)** Determination of the optimal soft threshold in WGCNA.

**Figure 3:**
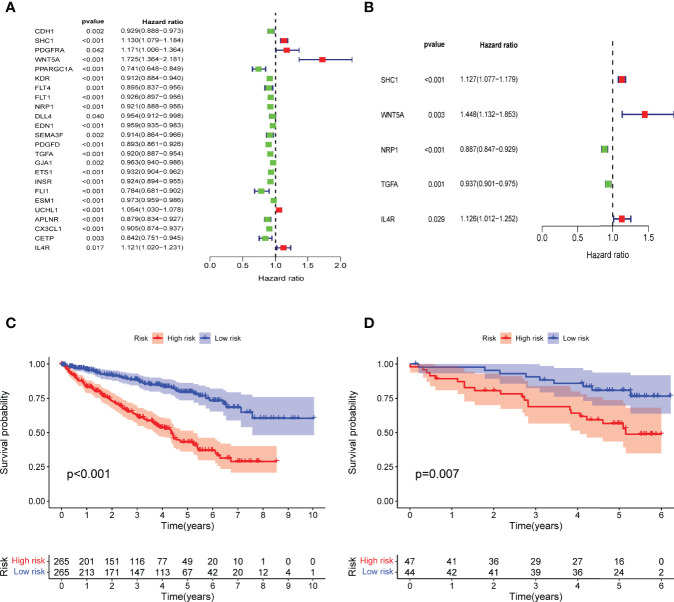
Construction of IRPDGs and prognosis of different IRPDGs subgroups. **(A)** Univariate Cox analysis of 24 immune-related hub genes. **(B)** Five genes are independently and significantly associated with ccRCC prognosis. **(C)** K–M survival analysis of the IRPDGs subgroups in the TCGA cohort. **(D)** K–M survival analysis of the IRPDGs subgroups in the ICGC cohort.

We screened a total of 9459 DEGs from the TCGA cohort (539 cancer samples, 72 paracancerous samples). The list of the immune-related genes was obtained from InnateDB and ImmPort databases. We obtained 946 immune-related DEGs by taking intersections (**Figures 2A, B**). Then, we performed functional enrichment analysis and identified 2137 GOs and 87 KEGG-related pathways (**Figures 2C, D**). Circle diagrams and specific tabular data are shown in **Figure S1** and **Table S1**. Candidate genes (n = 946) were then subjected to WGCNA. Logarithm log(K) had nodes with connectivity K and negatively correlated with logarithm log(P(K)) of node probabilities, with a correlation coefficient greater than 0.9. Based on the relationship between soft-threshold power and average connectivity, the optimal power for scale-free network-based networks was determined as 3. Seven modules were finally settled. Based on Pearson correlation coefficients between the modules and sample features, brown and green modules closely related to ccRCC were selected, and the top 40 core genes were identified for a follow-up study by CytoHuba (**Figures 2E–G**). The Kaplan–Meier survival analysis and univariate Cox analyses identified 24 immune-related hub genes that were closely associated with RCC survival (**Figure 3A**, **Figure S2**).

### Construction of the IRPDGs and Clinical Variability Analysis

Multivariate Cox regression analysis of the 24 immune-related hub genes finally identified 5 independent prognostic genes (SHC1, WNT5A, NRP1, TGFA, IL4R) that significantly affected ccRCC survival (**Figure 3B**). We then constructed prognostic models for the IRPDGs with the following formula: IRPDGs = expression value of SHC1 * (0.12) + expression value of WNT5A * (0.37) + expression value of NRP1 * (−0.12) + expression value of TGFA * (−0.06) + expression value of IL4R * (0.12). The high-risk versus low-risk IRPDGs subgroups were defined based on the median risk score as risk threshold. The results showed that patients in the low-risk subgroup had a better prognosis than those in the high-risk subgroup (P < 0.001, log-rank test) (**Figure 3C**). Using ICGC data as external validation yielded results consistent with those of the TCGA dataset, both with P-values < 0.05 (**Figure 3D**).

**Figure 4:**
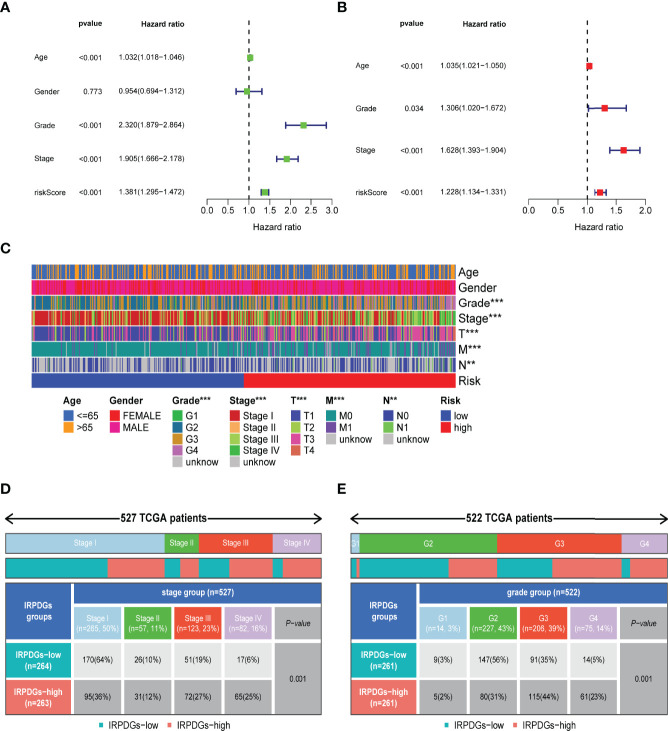
Differences in clinicopathological information and prognostic assessment in different IRPDGs subgroups. **(A)** Univariate Cox analysis of clinicopathological factors and IRPDGs (P < 0.05). **(B)** Multivariate Cox analysis of clinicopathological factors and IRPDGs (P < 0.05). **(C)** Distribution of clinicopathological features in the subgroups of IRPDGs in the TCGA cohort (** P < 0.01, *** P < 0.001). **(D)** Differential tumor stages in the IRPDGs subgroup of the TCGA cohort. **(E)** Differential tumor grades in the IRPDGs subgroup of the TCGA cohort.

The univariate Cox regression analysis including clinical and pathological information showed that age, IRPDGs, grade, and stage were high-risk factors associated with ccRCC prognosis. Further multivariate Cox analysis revealed that the IRPDGs remained an independent prognostic marker after adjusting for other factors (**Figures 4A, B**). The distribution of clinicopathological characteristics of the TCGA patient cohort across the IRPDGs subgroups is shown in **Figure 4C**, with detailed results in **Table S3**. **Figures 4D, E** specifically demonstrate the significant differences in the distribution of tumor stage and grade between the two risk subgroups.

### Molecular Characterization in the IRPDGs Subgroups

**Figure 5:**
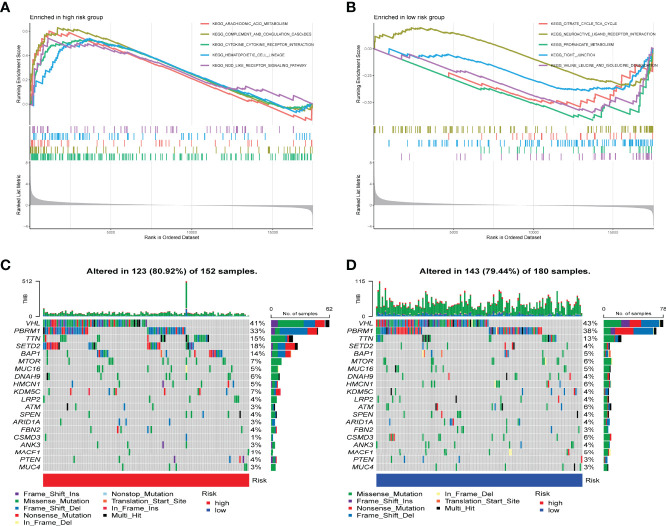
Molecular characterization of different subgroups of IRPDGs. **(A)** GSEA of the high-risk subgroup of IRPDGs. **(B)** GSEA of the low-risk subgroup of IRPDGs. **(C)** Significant gene mutations in the high-risk subgroup of IRPDGs. **(D)** Significant gene mutations in the low-risk subgroup of IRPDGs. The percentage of mutations is shown vertically on the right and the total number of mutations is shown above. The color-coding indicates the mutation type.

Molecular pathways and potential mechanisms enriched in the IRPDGs subgroups were identified by the GSEA. Pathways related to arachidonic acid metabolism and cytokine–cytokine interactions were enriched in the IRPDGs-high subgroup, while citrate–tricarboxylic acid (TCA) cycle and neuroactive ligand-receptor interaction pathways were enriched in the IRPDGs-low subgroup (**Figures 5A, B**). The detailed results of the GSEA are presented in **Table S2**. To further explore the immunological properties, we analyzed the somatic mutations between the subgroups. The number of mutations was higher overall in the high-risk subgroup than in the low-risk subgroup of the IRPDGs. Of these, the frequency of missense mutations was the highest, followed by shift deletions and nonsense mutations. **Figures 5C, D** shows the top 20 genes with the highest mutation rate in both subgroups. The mutation rate in VHL was higher than 40% in both subgroups. BAP1 and SETD2 mutation rates differed most between the two subgroups and were mostly seen in the high-risk subgroup.

### Immunological Characteristics Among the IRPDGs Subgroups

**Figure 6:**
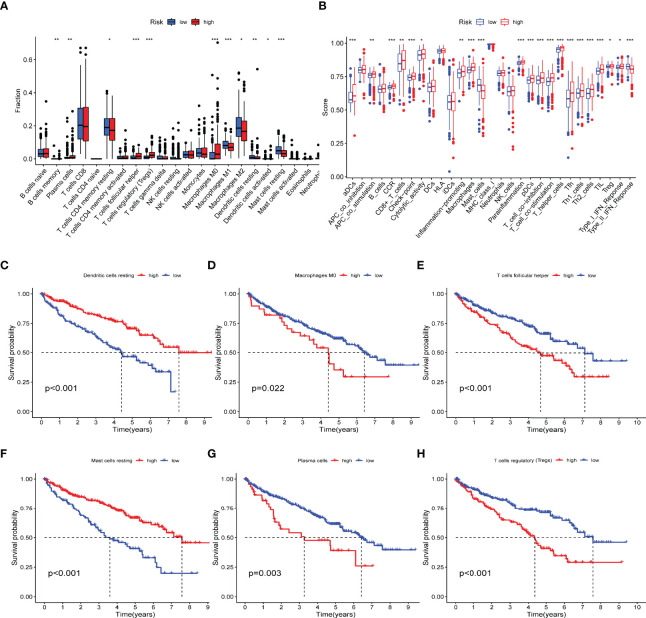
Immunological profiling of different subgroups of IRPDGs. **(A)** Differences in the distribution of tumor-infiltrating immune cells in TMEs of different IRPDGs subgroups. The difference between the two groups was statistically significant (ns: not significant, * P < 0.05, ** P < 0.01, *** P < 0.001). **(B)** Distribution of immunerelated functional indicators in different subgroups of IRPDGs. The difference between the two groups was statistically significant (ns: not significant, * P < 0.05, ** P < 0.01, *** P < 0.001. **(C–H)** Six tumor-infiltrating immune cells significantly associated with ccRCC prognosis.

The CIBERSORT is an analysis tool developed in Java and R for characterizing cell composition from tissue gene expression profiles. It uses a deconvolution algorithm to estimate the abundance of member cell types in mixed cell populations based on gene expression data (34). Here, we use CIBERSORT to explore the proportion of various TIICs in the different IRPDGs subgroups. The high-risk subgroup was mainly rich in Tfh, Tregs, plasma cells, and M0 macrophages; the low-risk subgroup had more M1 macrophages, dendritic cells resting, and mast cells resting (**Figure 6A**). The ssGSEA was applied to explore indicators of immune function involved in the two risk subgroups. The results showed that the IRPDGs-high subgroup was more enriched in CD8+ T cells, cytolytic activity, promotion of inflammogenesis, T cell co-inhibition, and costimulation, and type I IFN responses; in contrast, the IRPDGs-low subgroup was enriched in mast cells and functions related to type II IFN responses (**Figure 6B**). We then explored the value of the abovementioned differential TIICs for prognosis and found that patients with higher expression scores for mast cells resting and dendritic cells resting had a better prognosis, while those with high expression scores for plasma cells, follicular helper T cells, Tregs, and M0 macrophages had a worse prognosis. This was consistent with the prognostic model results that the low-risk subgroup of IRPDGs had a better prognosis (**Figures 6C–H**).

### Survival Analysis Between the IRPDGs Subgroups

**Figure 7:**
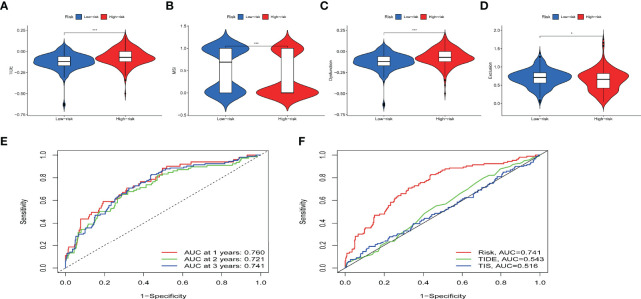
The value of IRPDGs in predicting ICI treatment response and prognosis. **(A–D)** TIDE, MSI, and T cell exclusion and dysfunction score in different IRPDGs subgroups. The scores were compared using the Wilcoxon test (ns: not significant, *P < 0.05, ***P < 0.001). **(E)** IRPDGs predict overall survival at 1, 2, and 3 years for ccRCC treated with ICI. **(F)** Advantages of IRPDGs over TIDE and TIS for prognostic prediction.

Immunotherapy efficacy between the subgroups of IRPDGs was assessed using TIDE. A higher TIDE score is associated with poorer ICI therapy outcomes and poorer patient survival (38). Calculation of TIDE score for the IRPDGs subgroups showed higher TIDE scores in the high-risk subgroup than in the low-risk subgroup. This means that patients with low IRPDGs scores have a greater benefit from immunotherapy. In addition, the IRPDGs-low subgroup had a higher MSI and lower T-cell rejection and T-cell dysfunction, with p-values less than 0.05 for all of the outcomes (**Figures 7A–D**). Using the ROC analysis to evaluate the model for predicting disease progression, we showed that our model was able to predict the survival rates of ccRCC patients at 1, 2, and 3 years with good accuracy and that the predictive value of the IRPDGs for overall survival (OS) was significantly better than that of TIDE or TIS. The AUC for IRPDGs, TIDE, and TIS was 0.741, 0.543, and 0.516, respectively (**Figures 7E, F**).

## Discussion

Considering that ccRCC is an immunogenic and vascularized tumor (27), ICI therapy has shown great potential and has been approved by the US Food and Drug Administration (FDA) as the standard treatment for patients with early and advanced metastatic ccRCC (4, 8). However, the ORR to ICI therapy remains low, with only a small proportion of patients achieving long-term, sustained outcomes after the treatment. The combination of ICIs and TKIs is now increasingly studied and is superior to ICI monotherapy in terms of OS and ORR, but it is also associated with greater toxicity. Screening patients with potentially greater benefit from immunotherapy is necessary. The TME consists of tumor cells and other nonmalignant cells, including the surrounding TIICs, fibroblasts, and stromal cells (39, 40). Among them, TIICs regulate tumor development at different stages (41). We have knowledge from previous studies that ccRCC patients treated with ICI show different treatment responses probably due to the heterogeneity of TME (30). Therefore, an in-depth investigation of the interaction mechanisms and biological functions of TIICs in the TME can help to optimize current immunotherapy. Given the limited assessment tools available, new biomarkers that effectively predict the clinical response and prognosis of ICI therapy are urgently needed to achieve precision immunotherapy.

A total of 946 immune-related DEGs were identified in our study. The final IRPDGs was constructed based on the five independent prognostically significantly associated genes and was validated in the ICGC dataset. After adjusting for clinical and pathological variables, the IRPDGs remained an independent prognostic factor. The five genes used to construct the IRPDGs were WNT5A, NRP1, TGFA, SHC1, and IL4R. The Wnt family member 5A (WNT5A) is a classical noncanonical WNT ligand that belongs to a large family of WNT cysteine-rich secretory glycoproteins and is involved in multiple signaling pathways that regulate a variety of cellular processes (42, 43). WNT5A mediates the activation of Tregs and tumor-associated macrophages (TAM), which thereby induces immunosuppression during the progression of castration-resistant prostate cancer (44). WNT5A may be a potential target for the prevention of gastrointestinal radiotoxicity because its absence impairs the ability of individual intestinal stem cells (ISCs) to form crypt spheroids after irradiation, whereas the addition of WNT5A saves ISCs from radiation-induced cell death (45). Tumor angiogenesis is considered an important phenomenon in the progression of tumors and is associated with overexpression of Neuropilin1 (NRP1) receptors. NRP1 is a nonenzymatic transmembrane glycoprotein, and recent studies have shown that high NRP1 surface expression leads to more suppressive Tregs and reduced progression-free survival (PFS). NRP1 is an independent novel marker in RCC (46), which forms a complex with transexpressed VEGFR2 and inhibits tumor angiogenesis, thereby improving patient survival. NRP1 is also highly expressed in glioblastoma multiforme (GBM) and is closely associated with the prognosis of GBM patients (47). As a member of the epidermal growth factor family, TGFA binds to EGFR to activate a series of signaling pathways that regulate cell biological processes such as cell proliferation, migration, differentiation, and energy metabolism (48, 49). TGFA also promotes cell proliferation and epithelial-mesenchymal transition (EMT) in prostate, liver, and breast cancers (50–53). SHC1 is a kind of skeletal protein that is associated with cell membrane metabolism and exerts its regulatory role mainly through the EGFR pathway (54, 55). Overexpression of SHC1 is associated with poor survival in stage IIA colon cancer (56). SHC1 encodes a splice protein that acts on various tyrosine kinase signaling pathways and is thought to be a key mediator in promoting immunosuppressed breast cancer (55). Interleukin 4 receptor (IL4R) is a type I cytokine receptor produced by activated Th2 cells and mast cells. The IL-4/IL-4R axis plays a role in immunity and inflammation, and IL4R has been shown to interact with SHC1 (57, 58).

Our survival analysis revealed that patients in the low-risk subgroup of the IRPDGs had a better prognosis. We further explored the prognostic characteristics of ccRCC patients in the IRPDGs subgroups using TIDE. Peng Jiang established the online TIDE score combining T cell dysfunction and T cell exclusion characteristics, which predicts immunotherapy responsiveness by calculating genome-wide expression profiles of pre-treatment patients, simulating tumor immune escape with different levels of CTLs (38). TIDE has shown outstanding advantages over other biomarkers for the prediction of ICI therapy responsiveness in prospective clinical trials. Our results showed higher TIDE scores and lower T-cell dysfunction and T-cell rejection in the high-risk subgroup of the IRPDGs. This suggests that patients in IRPDGs-high groups had more immune dysfunction and were less responsive to immunotherapy. In contrast, the IRPDGs-low group with a lower TIDE score and higher MSI score had a greater benefit from immunotherapy and a better prognosis. Interferon-gamma-7–related 18-gene T-cell inflammatory signaling (TIS) is a good predictor of ICI therapy reaction and prognosis in a variety of tumors (37, 59, 60). Through the analysis of the ROC curve, we found that the prediction value of total survival time was significantly better for the IRPDGs than for TIDE and TIS, with the AUC of IRPDGs, TIDE, and TIS being 0.741, 0.543, and 0.516, respectively. This means that the IRPDGs can effectively identify high-risk patients and select those who are more suitable for immunotherapy. Moreover, the IRPDGs, which consist of only five genes, are also easier to detect.

The TME plays a crucial role in the tumors’ growth and migration. The GO enrichment and KEGG pathway analyses of the immune-related DEGs revealed a variety of immune-associated pathways and functions, including leukocyte-mediated adaptive immunity, cytokine–cytokine receptor interactions, chemokine, T-cell receptor, and B-cell receptor signaling pathways. Based on the results above, we further explored the mechanisms of TIICs in ccRCC immunomodulation through the GSEA. Our study showed large differences in the composition of TIICs between the IRPDGs subgroups. Six TIICs that differed significantly between the subgroups were significantly associated with the survival of patients with ccRCC. Tfh, Tregs, plasma cells, and M0 macrophages were more abundant in the IRPDGs high-risk subgroup, while M1 macrophages, mast cell resting, and dendritic cell resting was more frequently seen in the low-risk subgroup. As one of the important immunosuppressive cell types, Tregs are highly immunosuppressive to effector cells. It has been confirmed that with the development of ccRCC, the infiltration of Treg cells increases, which indicates a poor prognosis (61, 62); and the degree of Treg infiltration is lower in some patients treated with ICI to CR (63). Previous studies have suggested that the infiltration of ccRCC by memory B cells and Tfh indicates a poor prognosis; in contrast, dendritic cell resting, mast cell resting, and eosinophilia are significantly associated with improved prognosis (64). Stefanie Regine Dannenmann et al. showed that pro-inflammatory macrophage phenotype (M1) is induced by IFNγ and lipopolysaccharide (LPS), and some factors associated with M1 correlate with prolonged survival in ccRCC (65). Our findings support these conclusions. Further exploration of the molecular immune functions between the IRPDGs subgroups revealed that the high-risk subgroup was more likely to involve CD8+ T cells and tumor-infiltrating lymphocytes (TILs), whereas the low-risk subgroup involved more mast cells and type II IFN responses. CD8+ T cell infiltration is associated with longer PFS and OS in most solid tumors (66); in contrast, in ccRCC, infiltration by CD8+ T cells predicts a poor prognosis (67–69). Type II interferon IFN-γ is released by T cells, NK cells, and macrophages. A recent study has explored the relationship between PD-L1 expression and IFN-γ by RT-PCR and western blot analysis; it showed that PD-L1 was induced by typical IFN-γ signaling in ccRCC cell lines, and IFN-γ-positive tumor tissues had high levels of PD-L1-mRNA expression and showed better OS (70). These findings further supported that the low-risk subgroup of the IRPDGs had better prognosis and immune efficacy than the high-risk subgroup.

A prerequisite for the efficacy of ICI is the generation of immunogenic neoantigens by tumor-specific mutations and the promotion of immune infiltration. Tumor mutational load (TMB) is an important predictor of ICI efficacy in many malignant tumors (71). We explored differences in somatic mutations between the subgroups and showed that missense mutations occurred most frequently between the two subgroups, followed by shift deletions and nonsense mutations. Missense mutations are associated with increased tumor antigen presentation and CD8+ T cell infiltration in most of the solid tumors (61, 72, 73). The VHL mutation rate was higher than 40% in both IRPDGs subgroups, and genes with the largest mutational differences were SETD2 and BRCA1-associated protein-1 (BAP1), which accounted for 18%:4% and 14%:5%, respectively. SETD2 is a histone methyltransferase whose normal function is of great importance in genome stability and DNA damage repair (74, 75). SETD2 mutations are associated with shorter PFS and OS in metastatic RCC and breast cancer (76). BAP1 is a kind of deubiquitinase that regulates various cellular functions including proliferation, differentiation, and metabolism (77, 78). BAP1 is mutated in lung cancer, thyroid cancer, kidney cancer, melanoma, and mesothelioma (79–81). Previous studies have found that the rate of BAP1 mutation increases with the increase in the ccRCC stage. BAP1 and SETD2 mutations are associated with disease progression and indicate a worse prognosis (82, 83). These data are consistent with our survival results; namely, patients with high SETD2 and BAP1 mutations in the IRPDGs-high subgroup had a poorer prognosis than those in the low IRPDGs subgroup.

In conclusion, with the advent of the era of accurate immunotherapy, IRPDGs shows obvious advantages in evaluating the response to ICI treatment and in predicting the prognosis. TIICs, which are significantly related to the prognosis of ccRCC in the IRPDGs subgroups, are helpful to guide future immunotherapy. The IRPDGs is a promising tool to guide clinical practice. More in-depth studies are needed to further confirm this point.

## Data Availability Statement

The raw datasets used in this study can be found in online databases, respectively, which are described in details in Methods sections. The source data and codes for reproducing the results and figures are freely accessible from https://www.jianguoyun.com/p/Df1PX6AQq5usChjNyrAEIAA.

## Author Contributions

QW: Research design and drafting of manuscripts. HT, XLu, and JC: Data analysis. XZ, XLi, YC, and YL: Literature review. YX, SH: Reviewing, revising, and guiding the writing of the manuscript. All authors contributed to the article and approved the submitted version.

## Funding

This study was funded by the Shaanxi Provincial Innovation Capacity Support Program (Approval No. 2018TD-002).

## Conflict of Interest

The authors declare that the research was conducted in the absence of any commercial or financial relationships that could be construed as a potential conflict of interest.

## Publisher’s Note

All claims expressed in this article are solely those of the authors and do not necessarily represent those of their affiliated organizations, or those of the publisher, the editors and the reviewers. Any product that may be evaluated in this article, or claim that may be made by its manufacturer, is not guaranteed or endorsed by the publisher.
