# Supplementary material for: Immune-Associated Gene Signatures Serve as a Promising Biomarker of Immunotherapeutic Prognosis for Renal Clear Cell Carcinoma
Source: Front Immunol. 2022 May 24;13:890150. doi: 10.3389/fimmu.2022.890150 (PMC9171405; doi:10.3389/fimmu.2022.890150)
Supplement: Supplementary file 5 [file Table_3.docx]

| **Table S3. Clinicopathological Characteristics of the TCGA Cohort in Different IRPDGs Subgroups** | | | |
| --- | --- | --- | --- |
| **Variable** | **IRPDGs-low** | **IRPDGs-high** | ***P-*value** |
| Age |  |  |  |
| <60 | 124(47%) | 121(46%) | 0.862 |
| >=60 | 141(53%) | 144(54%) |  |
| Gender |  |  |  |
| Female | 102(38%) | 84(32%) | 0.122 |
| Male | 163(62%) | 181(68%) |  |
| Grade |  |  | 0.001 |
| Grade 1 | 9(3%) | 5(2%) |  |
| Grade 2 | 147(56%) | 80(31%) |  |
| Grade 3 | 91(35%) | 115(44%) |  |
| Grade 4 | 14(5%) | 61(23%) |  |
| Tumor stage |  |  | 0.001 |
| Stage I | 170(64%) | 95(36%) |  |
| Stage II | 26(10%) | 31(12%) |  |
| Stage III | 51(19%) | 72(27%) |  |
| Stage IV | 17(6%) | 65(25%) |  |
| Survival status |  |  | 0.001 |
| Alive | 221(83%) | 44(17%) |  |
| Dead | 143(54%) | 122(46%) |  |
